# Supplementary material for: Subgingival Microbiota of Mexicans with Type 2 Diabetes with Different Periodontal and Metabolic Conditions
Source: Int J Environ Res Public Health. 2019 Aug 31;16(17):3184. doi: 10.3390/ijerph16173184 (PMC6751498; doi:10.3390/ijerph16173184)
Supplement: Supplementary file 1 [file ijerph-16-03184-s001.pdf]

Table S1. Mean total individual levels (total DNA-probe count x 10^5) of 40 individual bacterial species subgingival plaque samples (N = 178).

	**PH non-T2D (*n* = 59)**			**PH T2D (*n* = 14)**			**GP non-T2D (*n* = 67)**			**GP T2D (*n* = 38)**		
	**Media**		**SEM**	**Media**		**SEM**	**Media**		**SEM**	**Media**		**SEM**
Total levels ‡	133.16	±	17.72	268.01	±	40.63	128.77	±	11.96	349.25	±	23.26
A. georgiae €	1.23	±	0.22	3.04	±	1.09	1.52	±	0.44	6.17	±	1.05
A. israelii €	4.11	±	0.57	10.41	±	5.05	4.59	±	0.77	10.91	±	1.92
A. naeslundii 1 €	7.65	±	1.19	13.65	±	2.76	6.79	±	1.21	13.69	±	2.10
A. odontolyticus	1.71	±	0.28	2.46	±	0.64	1.57	±	0.24	3.89	±	0.53
A. viscosus * €	6.26	±	1.13	14.48	±	4.28	7.18	±	1.31	13.08	±	2.22
A. actinomycetemcomitans €	0.65	±	0.12	1.79	±	0.80	1.13	±	0.22	2.16	±	0.24
C. gracilis †	1.05	±	0.20	3.45	±	0.70	0.74	±	0.11	6.10	±	1.07
C. rectus €	1.49	±	0.28	3.17	±	0.82	1.41	±	0.19	4.27	±	0.59
C. showae *	1.22	±	0.22	3.83	±	1.04	1.00	±	0.13	3.04	±	0.39
C. gingivalis	2.69	±	0.43	5.12	±	0.90	2.52	±	0.41	5.16	±	0.80
C. ochracea * €	1.62	±	0.41	3.84	±	1.42	1.06	±	0.17	5.65	±	1.16
C. sputigena †	2.21	±	0.39	6.30	±	1.62	1.52	±	0.20	6.75	±	1.10
C. matruchotii £	7.26	±	1.11	14.84	±	4.83	9.08	±	1.15	16.80	±	2.43
E. corrodens	4.95	±	0.86	7.82	±	1.34	2.98	±	0.45	7.74	±	1.13
E. saburreum † €	4.82	±	0.71	14.87	±	5.35	5.52	±	1.46	12.46	±	1.34
E. sulci	4.11	±	0.83	4.89	±	0.94	2.57	±	0.46	5.51	±	0.92
F. nucleatum €	3.42	±	0.64	8.21	±	2.27	3.95	±	0.63	10.58	±	1.24
F. periodonticum €	4.16	±	0.71	5.19	±	0.83	3.84	±	0.49	6.58	±	0.87
G. morbillorum	4.21	±	0.65	7.36	±	1.55	4.46	±	0.78	8.59	±	1.11
L. buccalis	3.35	±	0.45	6.95	±	1.41	5.86	±	1.75	13.56	±	2.75
N. mucosa *	3.27	±	0.62	6.97	±	0.78	1.90	±	0.33	8.77	±	1.66
P. micra €	3.39	±	0.57	6.65	±	1.91	3.77	±	0.80	14.10	±	2.12
P. endodontalis	0.54	±	0.11	1.68	±	0.48	0.31	±	0.08	0.99	±	0.16
P. gingivalis	3.36	±	0.68	4.12	±	0.86	8.04	±	1.10	10.88	±	1.18
P. intermedia	4.97	±	1.15	7.08	±	1.97	5.74	±	0.91	15.16	±	2.55
P. melaninogenica	4.13	±	0.76	5.85	±	1.01	3.24	±	0.57	10.15	±	1.24
P. nigrescens	4.07	±	0.74	7.06	±	1.42	3.51	±	0.42	15.92	±	2.03
P. acnes	1.49	±	0.30	3.50	±	1.31	1.08	±	0.26	4.44	±	1.10
S. artemidis * €	1.76	±	0.37	3.27	±	0.63	0.77	±	0.11	4.74	±	0.52
S. noxia	1.70	±	0.43	3.64	±	1.49	0.66	±	0.11	3.76	±	0.48
S. anginosus * €	1.93	±	0.35	5.84	±	1.26	1.56	±	0.26	8.58	±	1.10
S. constellatus † €	2.16	±	0.35	4.91	±	0.71	2.07	±	0.33	9.22	±	1.10
S. gordonii *	2.06	±	0.34	6.38	±	1.74	1.75	±	0.26	5.72	±	0.75
S. intermedius *	2.11	±	0.46	3.94	±	0.72	1.29	±	0.19	6.22	±	0.75
S. mitis	4.34	±	0.93	6.98	±	1.07	2.10	±	0.37	9.49	±	0.83
S. oralis	2.00	±	0.28	6.10	±	0.99	1.88	±	0.43	9.84	±	1.29
S. sanguinis † €	2.43	±	0.34	9.18	±	2.58	3.05	±	0.78	9.54	±	0.98
T. forsythia	11.40	±	2.48	7.64	±	1.70	10.53	±	1.89	9.55	±	1.33
T. denticola	2.90	±	0.53	5.27	±	1.16	3.76	±	0.44	5.63	±	0.83
V. parvula €	9.32	±	1.80	21.15	±	3.66	7.03	±	1.10	25.86	±	2.63

Paired differences were determined by Mann-Whitney U test: * $p < 0.05$, † $p < 0.01$ and ‡ $p < 0.001$. PH non-T2D vs. PH T2D; § $p < 0.05$, £ $p < 0.01$ and € $p < 0.001$ GP non-T2D vs. GP T2D. PH: Periodontal-health; GP: Generalized-Periodontitis; non-T2D: no diabetic individuals; T2D: Type-2-Diabetes; SEM: Standard Error of de Mean.

Table S2. Mean prevalence (% sites colonized) ± SEM of 40 individual bacterial species of subgingival plaque samples (*N* = 178).

	PH non-T2D (*n* = 59)			PH T2D (*n* = 14)			GP non-T2D (*n* = 67)			GP T2D (*n* = 38)		
	**Media**		**SEM**	**Media**		**SEM**	**Media**		**SEM**	**Media**		**SEM**
A. georgiae €	40.37	±	3.67	64.58	±	8.22	33.54	±	3.66	72.07	±	5.00
A. israelii €	50.97	±	3.73	70.16	±	5.95	45.37	±	3.72	74.68	±	4.52
A. naeslundii 1 *	69.05	±	3.34	92.32	±	2.17	46.20	±	4.34	81.08	±	3.41
A. odontolyticus	46.83	±	4.47	70.86	±	7.62	45.15	±	4.03	66.23	±	4.66
A. viscosus €	61.79	±	3.51	86.12	±	4.07	48.03	±	4.05	79.68	±	3.42
A. actinomycetemcomitans	25.69	±	3.46	49.56	±	8.28	33.31	±	2.88	54.60	±	4.74
C. gracilis €	24.35	±	2.99	59.78	±	8.43	21.60	±	2.40	65.44	±	4.36
C. rectus *	32.42	±	3.69	62.90	±	6.56	34.50	±	3.02	67.27	±	4.75
C. showae £	32.83	±	3.56	59.51	±	7.66	38.40	±	3.06	63.27	±	5.17
C. gingivalis	42.02	±	4.22	69.31	±	9.18	42.78	±	3.66	49.35	±	4.95
C. ochracea €	33.08	±	3.81	54.16	±	6.96	30.62	±	3.22	72.23	±	4.42
C. sputigena €	36.53	±	3.67	79.48	±	6.29	35.00	±	3.34	68.06	±	4.36
C. matruchotii	70.55	±	3.25	80.08	±	4.65	64.68	±	3.73	87.37	±	2.34
E. corrodens §	46.27	±	3.45	81.69	±	6.38	39.51	±	3.86	65.40	±	5.62
E. saburreum	59.03	±	4.65	72.81	±	6.87	43.06	±	4.88	66.54	±	4.79
E. sulci	47.59	±	4.17	63.24	±	7.83	43.40	±	4.05	55.38	±	5.04
F. nucleatum	46.49	±	4.91	70.64	±	5.23	47.71	±	3.91	74.39	±	3.62
F. periodonticum	38.18	±	4.60	68.09	±	8.08	51.73	±	3.49	67.08	±	5.14
G. morbillorum £	40.31	±	4.64	70.76	±	7.61	35.70	±	3.79	60.83	±	5.08
L. buccalis £	45.50	±	3.67	72.79	±	7.99	44.06	±	3.92	70.62	±	4.62
N. mucosa €	46.97	±	4.57	75.20	±	6.37	41.96	±	3.95	76.42	±	4.52
P. micra €	45.77	±	4.65	51.91	±	6.93	37.83	±	4.31	74.52	±	4.34
P. endodontalis £	29.94	±	4.46	46.81	±	9.17	21.12	±	2.59	40.61	±	4.78
P. gingivalis	41.47	±	4.29	66.48	±	6.32	63.89	±	3.54	78.85	±	3.83
P. intermedia €	30.14	±	3.14	55.88	±	6.12	42.48	±	3.06	80.67	±	3.43
P. melaninogenica €	40.66	±	4.20	79.13	±	5.24	46.60	±	3.48	75.70	±	4.35
P. nigrescens €	48.58	±	5.03	61.85	±	5.88	47.48	±	3.63	76.98	±	3.41
P. acnes €	34.02	±	3.32	65.08	±	7.07	33.07	±	3.21	74.55	±	4.43
S. artemidis * €	35.53	±	4.17	68.45	±	6.16	27.94	±	2.96	66.51	±	4.96
S. noxia €	29.82	±	3.31	55.87	±	6.57	29.56	±	2.85	64.24	±	5.10
S. anginosus €	33.83	±	3.34	60.54	±	6.82	35.02	±	3.66	73.86	±	3.84
S. constellatus	44.51	±	4.24	73.24	±	5.88	41.34	±	4.02	81.31	±	3.07
S. gordonii €	43.77	±	4.80	70.75	±	8.37	40.17	±	3.75	69.54	±	4.96
S. intermedius €	45.96	±	4.69	77.89	±	6.09	41.15	±	3.45	76.03	±	4.80
S. mitis €	47.95	±	4.48	84.36	±	5.07	32.05	±	3.26	83.77	±	2.20
S. oralis €	39.82	±	3.67	72.75	±	6.23	35.82	±	3.41	80.08	±	3.95
S. sanguinis €	46.11	±	3.85	79.90	±	3.91	42.73	±	3.63	76.35	±	4.08
T. forsythia €	38.56	±	3.96	60.22	±	6.77	55.94	±	3.15	78.30	±	3.08
T. denticola	42.49	±	4.65	68.81	±	6.81	51.07	±	3.84	60.36	±	4.52
V. parvula £	46.67	±	4.03	66.04	±	6.15	49.17	±	3.89	75.64	±	3.94

Paired differences were determined by Mann-Whitney U test: * $p < 0.05$, PH non-T2D vs. PH T2D; § $p < 0.05$, £ $p < 0.01$ and € $p < 0.001$ GP non-T2D vs. GP T2D. PH: Periodontal-health; GP: Generalized-Periodontitis; non-T2D: no diabetic individuals; T2D: Type-2-Diabetes; SEM: Standard Error of de Mean.

Table S3. Mean proportion (% total DNA-probe count) ± SEM of 40 individual bacterial species of subgingival plaque samples (*N* = 178).

	PH non-T2D (*n* = 59)			PH T2D (*n* = 14)			GP non-T2D (*n* = 67)			GP T2D (*n* = 38)		
	**Media**		**SEM**	**Media**		**SEM**	**Media**		**SEM**	**Media**		**SEM**
A. georgiae €	1.75	±	0.53	1.10	±	0.57	1.11	±	0.28	1.69	±	0.32
A. israelii	3.45	±	0.74	3.21	±	0.88	3.84	±	0.73	3.21	±	0.53
A. naeslundii 1	11.87	±	1.33	7.96	±	0.83	6.95	±	1.37	5.23	±	1.16
A. odontolyticus	0.91	±	0.12	0.66	±	0.09	1.94	±	0.47	0.83	±	0.09
A. viscosus	7.59	±	1.23	7.65	±	2.21	5.75	±	1.11	3.47	±	0.38
A. actinomycetemcomitans	0.35	±	0.08	0.44	±	0.16	0.91	±	0.24	0.52	±	0.07
C. gracilis €	0.46	±	0.08	1.00	±	0.22	0.45	±	0.09	1.32	±	0.23
C. rectus	0.83	±	0.16	0.93	±	0.19	0.85	±	0.16	1.06	±	0.15
C. showae	0.98	±	0.26	0.84	±	0.16	0.62	±	0.11	0.73	±	0.10
C. gingivalis	1.32	±	0.18	1.79	±	0.41	1.97	±	0.35	1.01	±	0.21
C. ochracea €	0.66	±	0.13	0.82	±	0.16	0.48	±	0.07	1.26	±	0.18
C. sputigena	0.99	±	0.12	2.67	±	0.72	1.07	±	0.15	1.55	±	0.23
C. matruchotii	8.03	±	0.83	5.80	±	1.03	7.85	±	0.83	6.04	±	0.84
E. corrodens	2.98	±	0.42	3.49	±	0.74	2.60	±	0.67	2.34	±	0.41
E. saburreum	6.06	±	1.14	5.20	±	1.26	3.50	±	0.71	2.86	±	0.27
E. sulci	2.70	±	0.48	1.59	±	0.36	1.56	±	0.30	1.40	±	0.30
F. nucleatum	2.54	±	0.39	2.47	±	0.52	3.68	±	0.80	2.73	±	0.32
F. periodonticum	1.76	±	0.26	1.58	±	0.24	3.01	±	0.62	1.61	±	0.20
G. morbillorum	3.36	±	0.81	3.20	±	0.71	2.97	±	0.54	2.03	±	0.28
L. buccalis	3.74	±	0.91	2.92	±	0.71	5.44	±	1.60	3.91	±	0.89
N. mucosa	2.49	±	0.47	3.74	±	0.71	2.03	±	0.38	3.29	±	0.70
P. micra £	2.42	±	0.44	1.65	±	0.30	2.24	±	0.42	3.43	±	0.51
P. endodontalis	0.48	±	0.17	0.36	±	0.12	0.33	±	0.09	0.20	±	0.03
P. gingivalis	2.43	±	0.61	1.49	±	0.32	9.07	±	1.05	4.49	±	0.61
P. intermedia	2.40	±	0.37	3.54	±	1.16	4.00	±	0.51	4.86	±	0.65
P. melaninogenica	1.83	±	0.24	2.27	±	0.34	2.92	±	0.52	3.16	±	0.60
P. nigrescens £	2.69	±	0.43	2.13	±	0.33	2.37	±	0.28	4.70	±	0.60
P. acnes	0.96	±	0.24	1.62	±	0.85	0.78	±	0.17	1.23	±	0.27
S. artemidis €	0.85	±	0.15	0.95	±	0.16	0.81	±	0.26	1.37	±	0.40
S. noxia £	0.54	±	0.07	0.82	±	0.19	0.57	±	0.15	0.91	±	0.12
S. anginosus €	1.21	±	0.21	2.13	±	0.75	0.98	±	0.18	1.98	±	0.19
S. constellatus €	1.49	±	0.33	1.95	±	0.53	1.50	±	0.31	2.57	±	0.32
S. gordonii	1.42	±	0.28	2.43	±	0.52	0.96	±	0.12	2.27	±	0.48
S. intermedius €	1.97	±	0.64	1.12	±	0.13	0.79	±	0.16	1.90	±	0.28
S. mitis €	3.16	±	0.62	2.82	±	0.54	1.39	±	0.43	2.76	±	0.28
S. oralis €	1.12	±	0.24	2.11	±	0.40	0.79	±	0.16	2.74	±	0.30
S. sanguinis £	2.05	±	0.36	3.17	±	0.68	2.05	±	0.43	2.70	±	0.34
T. forsythia	4.99	±	0.96	2.71	±	0.84	7.13	±	0.89	3.40	±	0.45
T. denticola	1.82	±	0.29	1.66	±	0.34	3.03	±	0.45	1.56	±	0.30
V. parvula §	5.30	±	0.86	6.96	±	1.40	4.64	±	0.66	6.69	±	0.65

Paired differences were determined by Mann-Whitney U test: PH non-T2D*vs*. PH T2D: Not significant; § $p < 0.05$, £ $p < 0.01$ and € $p < 0.001$ GP non-T2D vs. GP T2D. PH: Periodontal-health; GP: Generalized-Periodontitis; non-T2D: no diabetic individuals; T2D: Type-2-Diabetes; SEM: Standard Error of de Mean.
